# A Method for 3D Histopathology Reconstruction Supporting Mouse Microvasculature Analysis

**DOI:** 10.1371/journal.pone.0126817

**Published:** 2015-05-29

**Authors:** Yiwen Xu, J. Geoffrey Pickering, Zengxuan Nong, Eli Gibson, John-Michael Arpino, Hao Yin, Aaron D. Ward

**Affiliations:** 1 Department of Medical Biophysics, The University of Western Ontario, London, Ontario, Canada; 2 Robarts Research Institute, The University of Western Ontario, London, Ontario, Canada; 3 Department of Oncology, The University of Western Ontario, London, Ontario, Canada; Harvard Medical School, UNITED STATES

## Abstract

Structural abnormalities of the microvasculature can impair perfusion and function. Conventional histology provides good spatial resolution with which to evaluate the microvascular structure but affords no 3-dimensional information; this limitation could lead to misinterpretations of the complex microvessel network in health and disease. The objective of this study was to develop and evaluate an accurate, fully automated 3D histology reconstruction method to visualize the arterioles and venules within the mouse hind-limb. Sections of the tibialis anterior muscle from C57BL/J6 mice (both normal and subjected to femoral artery excision) were reconstructed using pairwise rigid and affine registrations of 5 µm-thick, paraffin-embedded serial sections digitized at 0.25 µm/pixel. Low-resolution intensity-based rigid registration was used to initialize the nucleus landmark-based registration, and conventional high-resolution intensity-based registration method. The affine nucleus landmark-based registration was developed in this work and was compared to the conventional affine high-resolution intensity-based registration method. Target registration errors were measured between adjacent tissue sections (pairwise error), as well as with respect to a 3D reference reconstruction (accumulated error, to capture propagation of error through the stack of sections). Accumulated error measures were lower (*p*<0.01) for the nucleus landmark technique and superior vasculature continuity was observed. These findings indicate that registration based on automatic extraction and correspondence of small, homologous landmarks may support accurate 3D histology reconstruction. This technique avoids the otherwise problematic “banana-into-cylinder” effect observed using conventional methods that optimize the pairwise alignment of salient structures, forcing them to be section-orthogonal. This approach will provide a valuable tool for high-accuracy 3D histology tissue reconstructions for analysis of diseased microvasculature.

## Introduction

The microvasculature constitutes the complex distal end of the vascular tree, the structure of which is vital to ensuring optimal delivery of oxygenated blood throughout the tissue. Microvascular structure is inherently 3D, as the vessels are arranged as a highly branched network that courses throughout the tissue. Understanding the microvessel network organization, and its rearrangement during pathology, could thus be critical to dissecting the basis of organ dysfunction during disease. A structural appreciation of the vessel wall components of the microvascular tree is also vital. This is particularly important for assessing arterioles and venules, vessels that are wrapped to varying extents by vascular smooth muscle cells. The smooth muscle layer of the vessel wall determines vascular tone and thus blood pressure and flow rate. Thickening of the smooth muscle layer can lead to hypertension [1] and the arrangement of the smooth muscle layers with respect to vessel density, organization, and circumferential wrapping are vital to downstream flow in capillary beds. [2]

Conventional 2D histology provides planar information on microvessels and is useful for identifying components of the vessel wall. However, 3-dimensional architectural information cannot be ascertained. Moreover, 2D assessment of the microvasculature may lead to misinterpretations, particularly in the setting of restructured microvasculature during disease, where vessel and network morphometry cannot be predicted. In contrast to large and medium-sized vessels that can be embedded and sectioned in specific directions, the orientation of arterioles and venules of the microvasculature cannot be determined from standard histologic sections. Because of this, branching, bifurcations, and tortuosity of microvessels are difficult to interpret in conventional histology sections and misleading interpretations could arise from the 2D assessment of complex 3D structures. Focal pathology (e.g. occurring at bifurcation points and subregions of the vessel wall) can be better detected in a 3D volume of reconstructed tissue. Thus, there is a need for a highly accurate 3D reconstruction [3] to visualize and measure the microvasculature architecture in normal and diseased conditions.

There are various modalities with which to image the vasculature with 3D spatial information; however, most lack the sensitivity to assess fine pathological perturbations. Traditional light microscopy imaging of histology sections provides detailed information on tissue components and structure in 2D at high resolutions, but lacks the 3D context necessary for assessing structural aspects of pathologies in the vasculature. [4] Micro-CT allows for the spatial visualization of the lumina of the vessels after injection of a contrast agent. The surrounding soft tissue components, such as the vessel walls and connective tissues, are poorly resolved using this imaging modality due to lack of soft tissue contrast and retention of the contrast agent within the lumina. Casting the vasculature provides excellent lumen detail but is limited by the fact that the casting agent does not always perfuse throughout the microvasculature, and depiction of the vessel wall and surrounding tissue components is not possible. [5] Confocal fluorescence microscopy imaging is a powerful tool but has a limited spectrum of molecules which can be visualized, according to probe availability. This modality is also limited by the field of view and depth of penetration of the probes into the tissue. [6]

3D histology reconstruction of the microvasculature has been explained in a few contexts and proven to be useful. Steiniger et al undertook a 3D histology assessment of the spleen vasculature to reveal the terminal microvessel nodules. However, the alignment procedure used was performed manually and was therefore subject to the accompanying labour and operator variability. [7] 3D confocal microscopy has been used to observe microvascular branching patterns in diabetic models, but the authors noted there was possible bias in the results due to the limited depth of field. [8] Also using 3D confocal microscopy, capillary vessels in the skeletal muscle have been evaluated for vessel tortuosity, orientation, and mean capillary length. However the authors highlighted that the problem of variable visualization of capillaries “is even more pronounced [in thick sections], because antibodies and dyes have to travel longer distance[s] from the boundary of the sample.” They noted that new strategies in microscopy would be useful. [9] 3D reconstructions of histological tissues may also aid in the validation of high resolution 3D imaging techniques.

Accurate 3D visualization of histology could allow for obtaining features of the vessel wall, the surrounding tissue features, and the inherent spatial configuration of the vasculature. The lack of 3D spatial context in 2D histology may lead to misinterpretations of several different aspects of the vasculature, including vessel angle, vessel size, and vessel wall thickness. 3D reconstruction of 2D histology sections renders these types of measurements readily available and unambiguous. In previous work, 3D histology reconstruction has been performed using section-by-section pairwise registrations optimizing a feature or image similarity metric for adjacent section pairs, assuming that co-registration of similar structures in the 2D space of the pairwise images yields an accurate reconstruction. [10] This assumption is challenged by the fact that adjacent sections sample different tissue, and an accurate 3D reconstruction may not result in the section-to-section alignment of structures having similar appearance, such as two adjacent cross sections of a blood vessel. This notion is clear when one considers the case of a blood vessel oriented non-perpendicularly to the tissue section; in a correct 3D reconstruction, on any adjacent pair of sections, the blood vessel cross sections will not be exactly aligned in the 2D space of the histology sections to account for the non-perpendicular angular direction of the vessel in 3D. In general, 3D reconstruction techniques must be designed to avoid forcing curved or non-section-orthogonal structures to be orthogonal to the tissue sections [3], also known as the “banana-into-cylinder” problem [11], in order to preserve the original orientation of the vasculature for accurate assessment.

The objective of this work was to design, implement, and evaluate a method for 3D reconstruction of 2D histology sections of mouse tissue that is sufficiently accurate to enable interpretation of 3D arteriolar and venular networks. To address the banana-into-cylinder problem, the reconstruction method uses a section pairwise landmark-based registration, where the landmarks were homologous nuclei that were bisected by the microtome blade during sectioning. This choice of landmark type is based on the insight that cell nuclei, approximately 5 μm in diameter [12], are unlikely to appear on more than two adjacent sections. We conjectured that the use of such landmarks may address the banana-into-cylinder problem due to their appearance on not more than two consecutive sections, and their lack of orientational bias. This is in contrast to, for instance, the use of vessel centerlines to define landmarks; vessels will appear on many consecutive tissue sections and their angles of orientation through multiple tissue sections are coherent and smoothly varying. In general, there is a lack of bias in the centroid to centroid vectors of bisected nuclei across adjacent slides, compared to multi-slice non-section-orthogonal objects (such as the previously described vessels) that are smoothly varying at the scale of the section thickness. Also, nuclei are roughly spherically symmetric, so arbitrary cuts through nuclei should yield section-orthogonal centroid-to-centroid orientations. We compared the reconstruction error of the proposed nucleus-based registration to that given by more conventional intensity based registration. The registration algorithms were evaluated based on reference standard homologous nucleus features on adjacent sections to determine registration accuracy.

## Materials and Methods

### 2.1 Animal model

The experiments were conducted on tissue samples of the upper one third tibialis anterior (TA) hind limb muscle from 11 wild type C57BL/J6 mice. In 5 of the 11 mice, tissue was collected two weeks after induction of hind limb ischemia by femoral artery excision; these samples were expected to contain regenerated vasculature. This particular muscle segment was selected because of the consistent development of microvessels of diverse caliber following hindlimb ischemia. The remaining 6 mice were not subjected to hind limb ischemia; these samples were expected to contain normal vasculature. There were between 9 and 14 serial sections obtained from each sample.

The mice were perfused with saline post-mortem to remove the red blood cells from the vessel lumina and then perfusion-fixed at physiological pressure with 4% paraformaldehyde. The tissues were processed and paraffin-embedded after harvesting, then cut into 7×5 mm blocks and sectioned at 5 μm. To visualize the smooth muscle distribution down to the level of the arterioles and venules, sections were immunostained with smooth muscle (SM) α-actin using the monoclonal antibody (DAKO, M0851), and bound primary antibody detected with horseradish peroxidase (HRP)-conjugated secondary antibody and 3,3'-Diaminobenzidine chromogen (DAB, Vector Laboratories, SK-4100). This marked the smooth muscle layer of the vasculature (resulting in the vessel walls being stained with a brown color) which is shown in S1 Fig. The tissue was then counter stained with hematoxylin, resulting in blue-stained nuclei. The stained sections were then imaged with a ScanScope CS (Aperio Technologies, Vista, CA, USA) bright field slide scanner, at 20× objective with the 2× magnification engaged, resulting in a 0.25 μm isotropic pixel size.

#### 2.1.1 Ethics Statement

All experiments in this study were approved by the Animal Care and Veterinary Service Committee at The University of Western Ontario (Protocol # 2010–244) and were carried out in accordance with their requirements. Surgeries were performed under isoflurane anesthesia.

### 2.2 Image registration and validation approach

**Fig 1:**
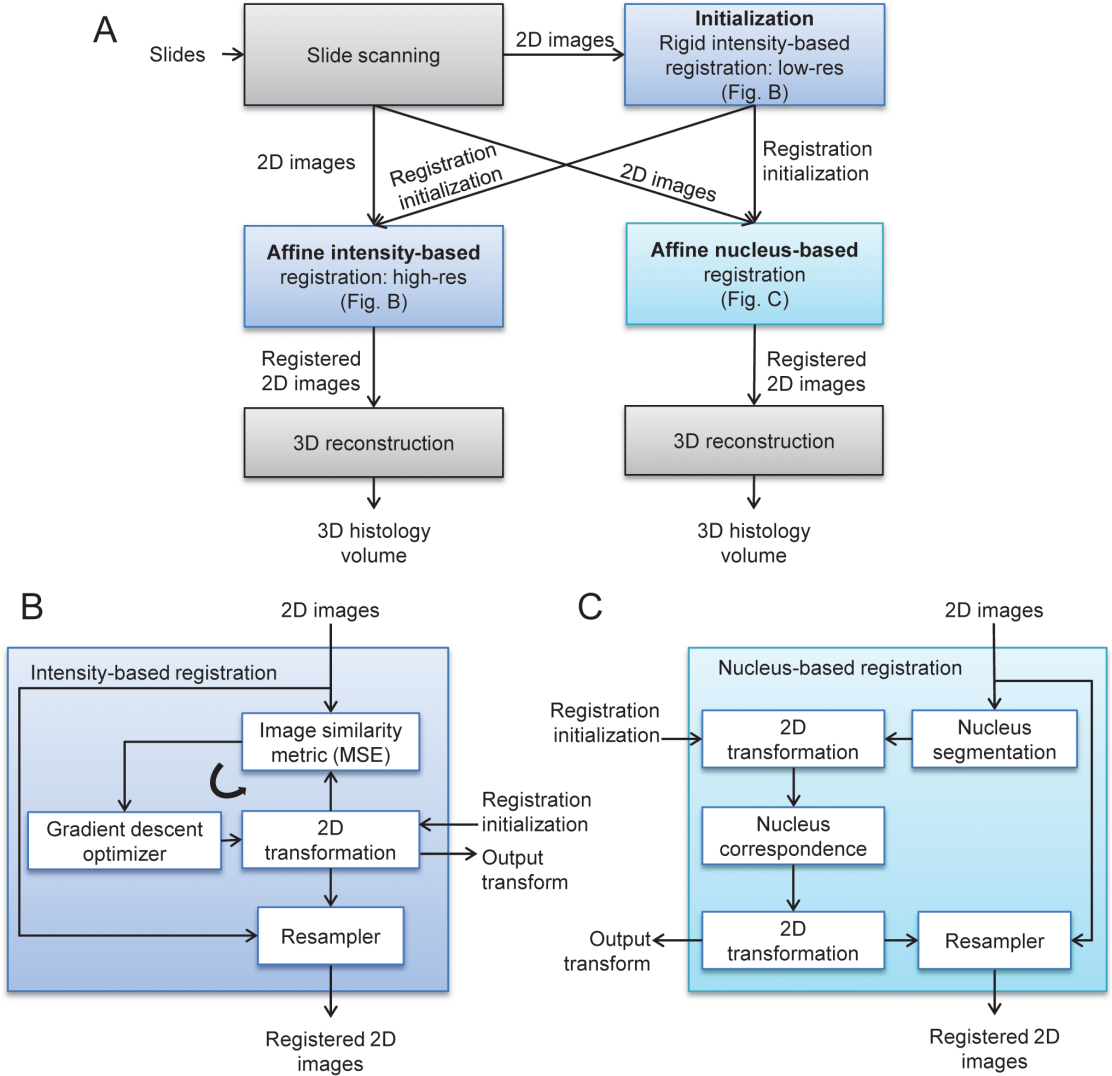
Diagram depicting the registration methods. The flow diagram in (A) depicts the overall experimental process, with (B) and (C) giving exploded views of the intensity-based and nucleus-based registration steps. Both approaches were initialized with a rigid, low-resolution intensity-based registration. The intensity-based registrations (B) were done using a standard iterative optimization loop. The nucleus-based registration (C) was computed non-iteratively in closed form based on automatically segmented and corresponded nucleus landmarks. Both methods were executed pairwise on each adjacent section pair, and as a final step these pairwise registrations were composed to form the final 3D reconstructed volume.

A high level overview of the methods is shown in Fig 1. All processing was performed using custom software developed in MATLAB 7.13 (The Mathworks Inc., Natick, MA, USA) except where otherwise indicated. Pairwise non-rigid affine nucleus landmark and intensity based registration was performed between serial sections of tissue in two dimensions and used to create a three dimensional volume. Two non-rigid affine registration methods were compared: high-resolution intensity-based registration using a mean squared error (MSE) image similarity metric, and the *affine nucleus landmark-based* registration that is the main contribution of this paper. A non-rigid affine registration involves rotation, translation, scaling and skew for non-rigid alignment of the moving image to the fixed image. The registrations were initialized with a rigid registration, which involves only rotation and translation of the moving image. MSE is the mean of the squared intensity differences between each pair of overlapping pixels in the fixed and moving comparison images. The ideal value of MSE is zero, and a gradient descent optimizer was used to find the optimal registration yielding an MSE closest to zero. Both methods are provided with the same initialization from a coarse, intensity-based rigid registration performed on low-resolution (downsampled) images using the MSE metric. This coarse 3D reconstruction was first performed via pairwise registration of adjacent tissue sections using an intensity-based registration, on low-resolution images (with extents of 172 × 264 pixels) obtained by downsampling using bilinear interpolation. This coarse registration yielded an initial alignment that was provided to both tested registration algorithms. For the landmark-based registration, nucleus landmarks were automatically extracted based on size and the hematoxylin stain color, and corresponded across adjacent sections according to similarity metric measures of the surrounding local image neighborhood. After pairwise adjacent section registration, the tissues were rendered into a 3D volume by a stacking process to visualize the histological vasculature.

### 2.3 Experimental methods

**Fig 2:**
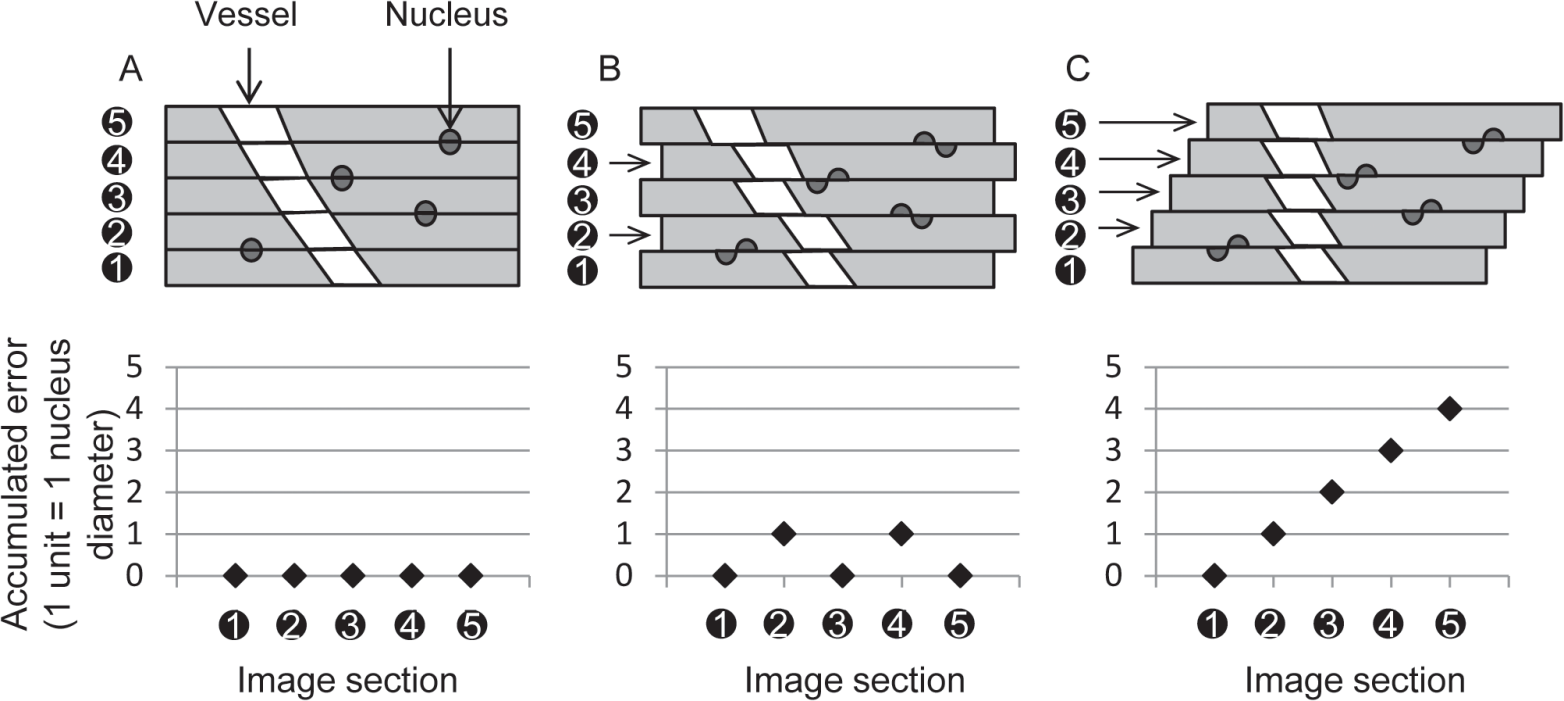
Comparison of the alignment of bisected nuclei when measuring the accumulated registration error. (A) The ideal error-free reference reconstruction, with bisected nuclei aligned with minimum residual error (a pairwise target registration error between corresponding halves of bisected nuclei of zero is depicted). (B) A reconstruction aligning nuclei with spatially unbiased error; but vessel connectedness (topology) and angle (geometry) are mostly conserved. (C) A reconstruction optimizing pairwise alignment of salient structures (the vessel cross sections in this example) preserves vessel topology but not geometry. Note that the pairwise target registration errors in (B) and (C) are similar, despite the lack of geometry preservation in C. The accumulated target registration error does capture the difference between (B) and (C); the plots in the bottom row indicate increasing accumulated error through the stack of sections.

A set of homologous *reference nucleus* landmarks were located with the segmentation algorithm. The landmarks were manually verified for accuracy, corresponded in pairs on adjacent sections, and used to evaluate the registrations. This set of nucleus landmarks was not used in registering the images (i.e. the reference landmarks were specifically excluded in the computations described in the Section 2.5). A *reference reconstruction* using these reference landmarks provides a surrogate for an ideal reconstruction (Fig 2(A)) that preserves both topology and geometry. Topology preservation maintains connectedness of structures and geometry preservation maintains the original positions and orientations of structures in the reconstruction.

Our need for high-accuracy reconstructions requires that we evaluate our method against a reference standard providing precision of < 10 μm, with accuracy measured throughout all regions of the tissue. These requirements preclude the use of a 3D reference image obtained using CT or MRI as these imaging modalities do not provide the necessary resolution and/or soft tissue contrast to resolve the necessary small, homologous point landmarks to measure reconstruction error throughout the spatial extents of the volume. Micro CT of contrast-enhanced vasculature could provide sufficient landmarking precision at vessel bifurcation points, but this would spatially concentrate reconstruction error measurements around these points, precluding error measurement throughout all other tissue regions.

An ideal reference against which to evaluate our reconstructions would be a set of dense and evenly distributed landmarks, localizable with the necessary precision and accuracy. As it would be impractical to introduce a set of extrinsic landmarks meeting these criteria, we turn to a close intrinsic surrogate: the small, highly localizable cell nuclei distributed throughout the tissue. Specifically, we manually localized the subset of nuclei that were bisected by the microtome blade; these nuclei appear on homologous points on adjacent tissue sections. By aligning these bisected nuclei on adjacent sections throughout the volume, we re-established the spatial tissue homology that was broken during the tissue cutting process, yielding a reference 3D reconstruction that depicts the geometric and topological configuration of the tissue before it was cut.

It is important to note that although the method for constructing the reference reconstruction and the method for performing automatic reconstruction are both based on nuclei, there are two important differences that justify the use of this validation approach. First, as is typical in landmark-based registration evaluation, the landmarks used for the reference reconstruction are not used by the registration algorithm. Thus, the algorithm is validated using a completely different set of landmarks than those used to perform the reconstruction. Second, the landmarks used for the reference reconstruction were all manually verified by an operator for truth of correspondence and localisation, whereas for the reconstruction process, the landmarks were fully automatically identified and not checked by an operator.

### 2.4 Intensity-based registration

The histology images were obtained from the Scanscope CS slide scanner with isotropic 0.25 μm pixels and had extents of 17135 × 26398 pixels. To support faster processing for the intensity-based registration only, these images were downsampled to obtain images with isotropic 4 μm pixels using bilinear interpolation, resulting in an image size of 1071 × 1650 pixels. These downsampled images were then converted into greyscale images by averaging of the RGB color channels.

Fig 1(B) describes the intensity-based registration algorithm we tested. The images were registered using custom C++ software built on the Insight Segmentation and Registration Toolkit Version 4.4.1 (ITK) [13]. For the low-resolution registration used as initialization, the itk::RegularStepGradientDescentOptimizer optimizer was tuned for scale differences in transformation parameters using its SetScales() function; scales were set to be 10^2^ and 10^–2^ for the rotational and the translational components of the transformation, respectively. The maximum (i.e. initial) and minimum (i.e. defining convergence criterion) step sizes were set to 4 and 0.1, respectively, using the optimizer’s SetMaximumStepLength() and SetMinimumStepLength() functions. For the high resolution registration, these parameters were adjusted to 0.01 and 0.001, respectively, to refine convergence to the local optimum found by the low-resolution registration. All Parameters were chosen on experimentation with a sample not included in this study and not tuned for the samples in the study. In the interests of computational efficiency, we used the MSE image similarity metric because this is a mono-modality image registration problem and tissue staining is anticipated to be consistent within a set of serial sections. The space of 2D rigid transformations was searched to minimize MSE using a regular step gradient descent optimizer initialized with zero rotation and translation. Non-rigid affine registration was applied after the rigid intensity based registration with the optimizer initialized using the rigid registration parameters. After optimization, MATLAB’s *imtransform* function was used to apply the resulting set of 2D affine spatial transformations. This mapped the full resolution RGB histology image of each section to that of its adjacent section, yielding a 3D reconstruction from the intensity-based registration.

### 2.5 Nucleus feature extraction, correspondence, and registration

Fig 1(C) describes our nucleus landmark-based registration algorithm. Cell nuclei were automatically extracted from the images based on combined criteria of color and size. To determine the color and size criteria to be used for nucleus extraction, we used a separate set of mouse hind limb tissues that were not used for the experiments reported in this paper. On this separate set of tissues, we manually delineated nuclei which were counter stained with hematoxylin with varying degrees of staining (S2 Fig). Based on these manual delineations, a threshold value of < 80% in the green channel of the red-green-blue (RGB) color space and an area range of 6 μm^2^–160 μm^2^ were determined as the criteria for defining nuclei. Both the color and area criteria needed to be met for detection of each nucleus; samples of extracted nucleus points are shown in S3 Fig. Debris can occur in the blank/white space of the microscope slide surrounding the tissue and can have similar color and size criteria as nuclei. This debris was automatically excluded as nuclei by determining whether the surrounding area had similar appearance to the measured slide background. The surrounding area is defined by a 5 μm disk-shaped morphological dilation seen in S3 Fig. The area was compared to a mean green channel of >95%, standard deviation of <4%, which were chosen according to the typical appearance of the clear glass slide regions. The centroids of the extracted nuclei were used as the *nucleus landmarks* for registration.

**Fig 3:**
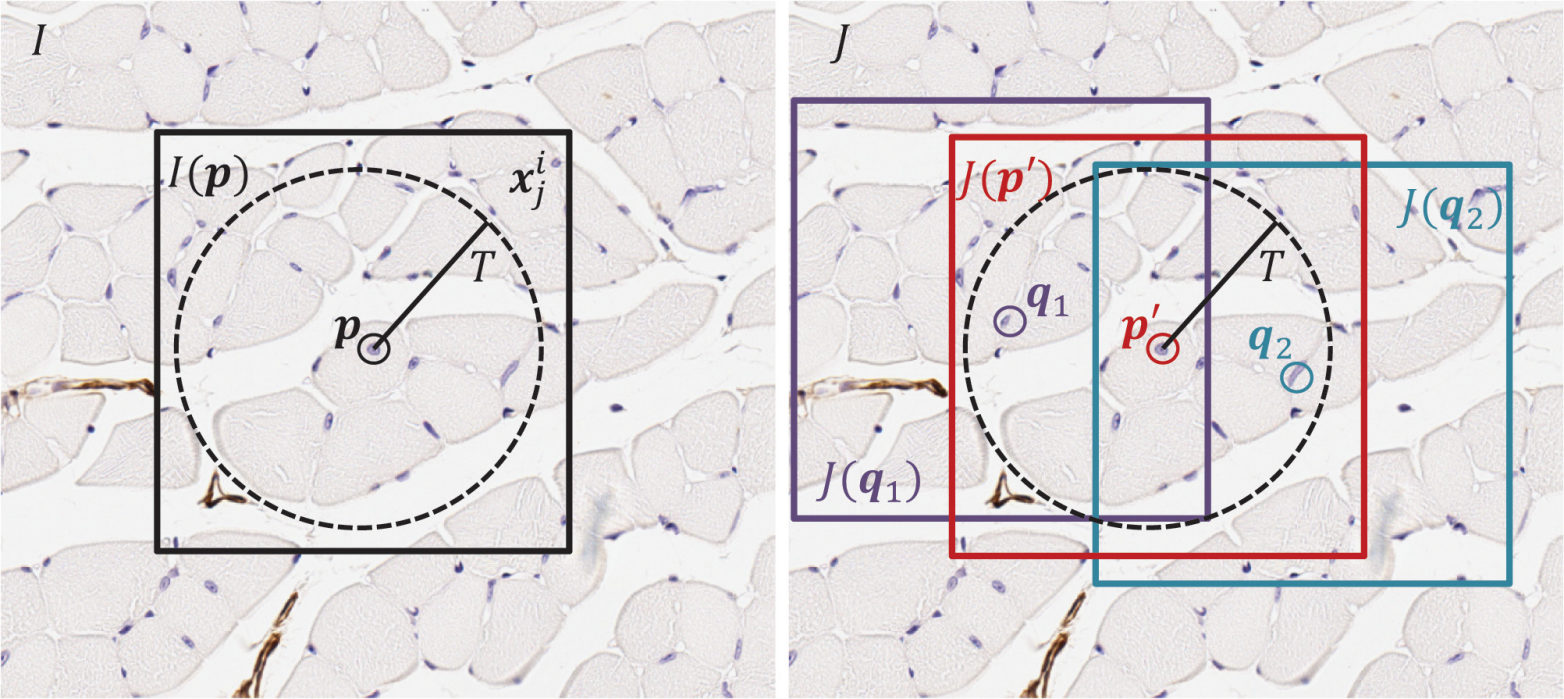
Nucleus correspondence method. An illustration depicting the approach to establishing correspondence of nucleus *p* in section *I* with its best matching nucleus in adjacent section *J*. In this example, the candidate nuclei on section *J* are *p’*, *q*_*1*_, and *q*_*2*_, lying within a dashed circle of radius *T* centred on *p* (only 3 of the 18 candidate nuclei within the circle are illustrated here for simplicity). The candidate nucleus with the most similar surrounding tissue appearance is selected to correspond to *p*. Surrounding tissue appearance similarity is measured using the *MSE* image similarity metric, comparing the local square region *I(p)* centered on *p* with the local square regions *J(p’)*, *J(q*_*1*_ *)*, and *J(q*_*2*_ *)* centered on the candidates *p’*, *q1*, and *q2*. In this example, since *MSE(I(p)*, *J(p’)) < MSE(I(p)*, *J(q1))* and *MSE(I(p)*, *J(p’)) < MSE(I(p)*, *J(q2))*, *p* is corresponded with *p’*.

Next, we estimated a correspondence between bisected homologous nuclei appearing on adjacent sections, in order to define an affine transformation registering each pair. Our approach to correspondence establishment is inspired by block matching-based image registration [14], and is based on the conjecture that for a given nucleus on a section, if a homologous nucleus exists within a local neighborhood on a neighboring section, it will be surrounded by tissue having similar appearance. All of the tunable parameters in our method were chosen based on experimentation with a sample not used in the study. Our approach to estimating correspondence for each detected nucleus involves defining a set of candidate nuclei within a defined local neighborhood on the adjacent section. An image similarity metric is then evaluated within a local region surrounding each candidate to find the candidate with the most similar surrounding tissue. A square local region was used to compare the neighborhoods of candidate nucleus landmark correspondences (Fig 3). The green channel of the square image was used and the window/level was adjusted (by software, without human interaction) to the window of 14 and level of 235 to enhance tissue-background contrast. The neighborhood image *I(p)* has a side length of 250 μm and surrounds each nucleus *p* on section *I*. We calculated the mean-squared error (*MSE* in the equation below) image similarity metric between *I(p)* and the local image neighborhoods *J(q)* surrounding all candidate nuclei *q* (within *T* = 100 μm of *p*) on adjacent section *J*. We corresponded each nucleus ***p*** on each section to the best matching nucleus ***p’*** on the adjacent section, with the best match defined as the one having the smallest *MSE* as defined above. Precisely,

$$p\prime =\underset{q\in \left\{x_{j}^{i},\forall i|D\left(p,x_{j}^{i}\right)<T\right\}}{\text{arg min}}\;MSE\left(I\left(p\right),J\left(q\right)\right)$$

where ***x*** ^*i*^_*j*_ is defined to be the *i*th nucleus on image *J* and *D* is defined to be the 2D Euclidean distance function. For each adjacent section pair, we defined a rigid transformation minimizing the residual error between the corresponding nuclei (i.e. the established ***p*** → ***p*** *’* correspondences) having the best (smallest) 100 *MSE* values found for that section pair. We retain only the best correspondences to define the registration since there will undoubtedly be many nuclei that do not have a homologous nucleus on the adjacent section, since not all nuclei are bisected by the microtome blade. The composition of these transformations, both rigid and affine, for each section pair yielded the 3D *nucleus landmark reconstruction*.

#### 2.5.1 Evaluation of registration accuracy

The registration accuracy was measured in terms of topology (i.e. preservation of structural connectivity) and geometry (i.e. preservation of distances and angles between structures). Accuracy was measured based on reference reconstructions of the tissues. For each sample, the reference reconstruction was determined by reference nucleus landmarks, which formed a separate set of corresponded nuclei not used to define the automated registration. To ensure the correctness of the reference reconstructions, every reference landmark correspondence was verified manually and any incorrect correspondences were discarded. We measured the *pairwise* registration error as the post-registration misalignment of reference nuclei on each section with the homologous corresponding reference nuclei on the next adjacent section. Thus, the pairwise registration error for a single nucleus is measured within the 2D spatial context of a section on which it appears. We also measured the *accumulated* registration error as the difference between the position of a reference nucleus given by a registration algorithm and its position within the reference registration. Thus, the accumulated registration error for a single nucleus is measured within the 3D spatial context of the reference reconstruction. This captures accumulation of error propagated through the series of pairwise section registrations. The pairwise error characterizes the performance of the reconstruction algorithm for each pairwise registration of adjacent sections independently. However, the accumulated error provides information about spatial bias in the pairwise error that may result in a reconstruction that is more erroneous than the pairwise error would suggest. This scenario is depicted in Fig 2. Although the scenarios depicted in Fig 2(B) and 2(C) have the same pairwise registration error and both preserve topology, the reconstruction Fig in 2(B) better preserves geometry and this is reflected in a lower accumulated registration error. Errors measured in these two spatial contexts are thus complementary, with the pairwise registration error capturing errors in topology of the reconstructed vasculature, and accumulated registration error capturing geometric errors.

Within the different spatial contexts described above, we measured two different types of registration error: the target registration error (TRE) and fiducial registration error (FRE). The “targets” and “fiducials” refer to reference points. Both the TRE and FRE are measured as the post-registration Euclidean distance between homologous fiducial pairs. Since, in an ideal registration, these homologous fiducial pairs should be perfectly aligned, the ideal value of TRE and FRE is zero. The difference between the FRE and TRE is in the choice of fiducials used. To calculate the FRE, the same nucleus landmarks that were used to define the registration are used to calculate the error. To calculate the TRE, the nucleus landmarks that were used to define the registration are not used to calculate the error; the separate set of reference landmarks, to which the algorithm is blinded, are used instead. Thus, the FRE provides sense of the best-case performance of the algorithm since the algorithm optimized the alignment of the same landmarks used to calculate the error, and the TRE provides a more realistic measure of performance. We evaluated the intensity-based and nucleus-based reconstructions separately by calculating the pairwise and accumulated TREs for each algorithm on each of both normal and regenerated mouse tissues. To determine the appropriate statistical tests for comparing the TREs generated by the different approaches, Kolmogorov-Smirnov normality tests were performed on the TRE distributions. We tested the null hypothesis that the median pairwise TRE of the intensity-based registration was the same as the median pairwise TRE of the nucleus-based registration using the non-parametric Wilcoxon sign rank test. A similar null hypothesis for the accumulated TRE was also tested.

We undertook several steps to give context to our measured registration errors. As a best-case estimate of fidelity of the reference reconstruction to a hypothetical ideal reconstruction, we measured the pairwise FRE of the reference reconstruction based on manually validated nucleus landmark correspondences. This measure reflects the amount of tissue distortion that was not compensated by our affine transformation model, which gives a sense of the discrepancy between the reference reconstruction and an ideal reconstruction. To provide context for interpretation of the pairwise TREs from the tested algorithms, we also measured the pairwise TRE using the reference nuclei using leave-one-out cross validation (LOOCV). Using this technique, one of the reference landmark pairs is removed (“left out”) and the registration is defined based on the remaining pairs. The registration error for the removed pair is calculated. This is performed N times for each of the N reference fiducial pairs, yielding N distinct TRE measurements which are averaged. Since the algorithm is only blinded to one reference landmark pair at a time, the pairwise TRE measured by LOOCV over the reference nuclei provides an optimistic measure of registration performance against which to compare the TRE calculated when the algorithm is blinded to all of the reference nuclei.

#### 2.5.2 Evaluation of image similarity metrics

Any observed differences the in error values observed between the intensity-based and nucleus-based reconstructions could be attributed to two main sources. The first is that the transformation yielding the MSE similarity metric optimum may not be coincident with the nucleus landmark-based transformation as defined by

$$T_{MSE}=\underset{T}{\text{arg min}}\;MSE\left(I,J\right),$$

where *I* and *J* are images of adjacent sections. The second is that the intensity-based registration optimizer could fail to converge to the desired optimum. If the optimum is not coincident with the landmark-based transformation, one could assert that the reconstructions given by the nucleus landmark-based approach would be different from those given by the intensity-based approach. This would be the case even with the use of a hypothetical ideal optimizer. If the intensity-based registration did not converge to the optimum, one could assert that the intensity-based approach using the MSE metric could perform equally as well as the nucleus landmark-based approach, given improvements to optimization. To gain insight into the reasons behind any such observed differences in error, we calculated the MSE image similarity metric in the spatial neighborhood of the transformations yielded by the nucleus-based reconstruction. This exploration could determine that the MSE optimum and nucleus landmark optimum are not coincident (i.e. if there exists a transformation with a lower MSE value than that given by the nucleus landmark transformation); in this case, a hypothetical ideal optimizer would not yield a reconstruction based on the MSE metric that would be equivalent to the nucleus-based reconstruction.

In this experiment, we explored the space of translations along the x and y directions separately, within ranges of 20 μm from the nucleus-based optimum, in increments of 0.5 μm. This experiment was performed on full resolution serial section images. For each of the x and y directions we found the optimal translation yielding the lowest MSE and recorded this value as a displacement from the nucleus landmark registration (i.e. a displacement of 0 μm reflects an MSE optimum coincident with a nucleus landmark registration optimum). We calculated the 95% prediction intervals of these displacements and recorded the upper bounds of these intervals to obtain an estimate of the expected translational offset between the nucleus based registrations and the registrations one could obtain via optimization of the MSE image similarity metric within the local neighborhood of the nucleus based registration. Under some assumptions (discussed in the penultimate paragraph of this paper), this allows a comparison between the nucleus-based reconstructions and reconstructions that could be achieved without the need for explicit localization and correspondence of nucleus landmarks (e.g. using robust optimization of MSE in a multi-resolution framework).

## Results

### 3.1 Vessel reconstruction properties

**Fig 4:**
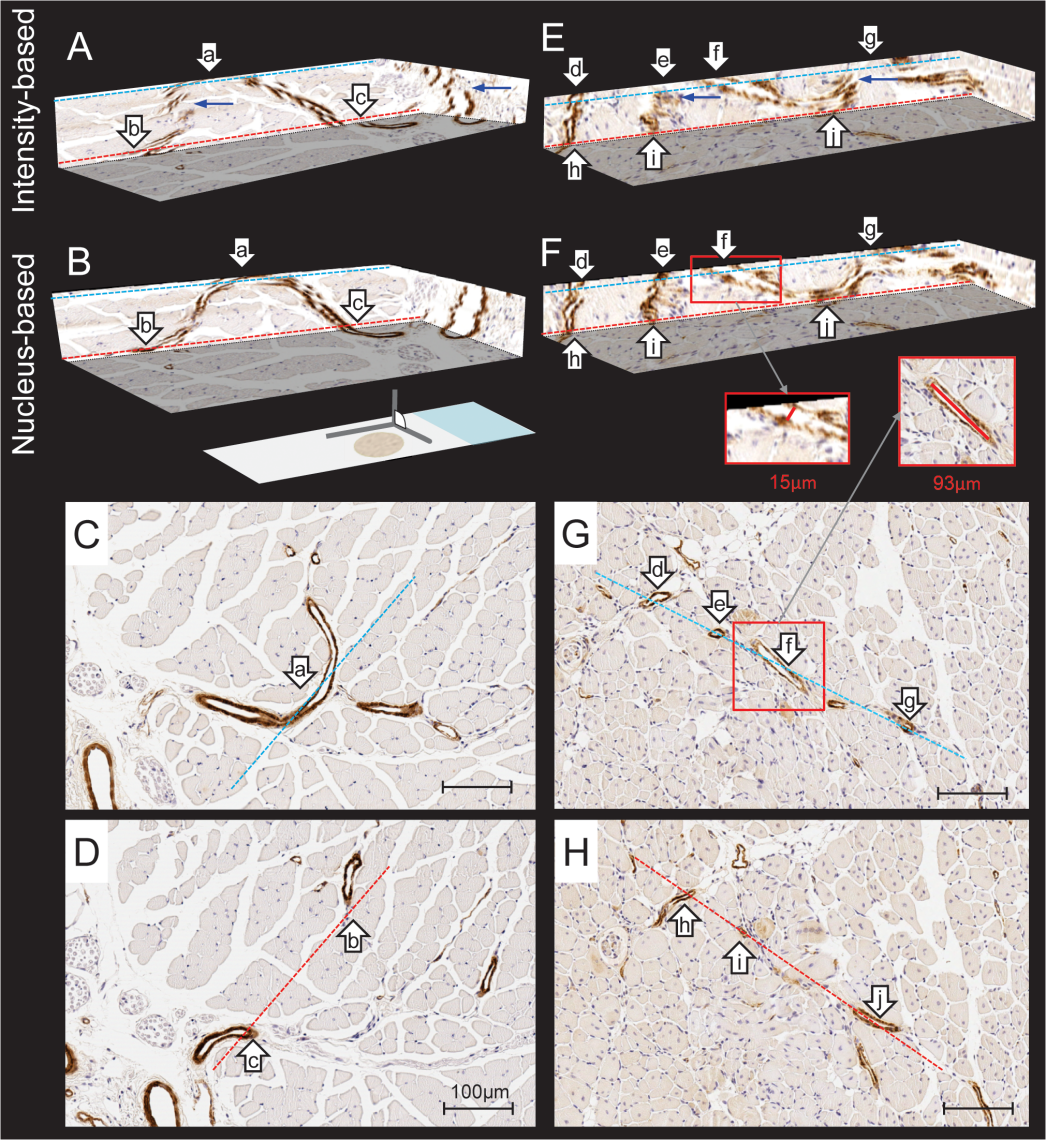
3D and 2D histology comparisons. 2D histology sections (pixel size 0.25 μm × 0.25 μm) and corresponding 3D reconstruction (voxel size of 0.25 μm × 0.25 μm × 5 μm) of serial histology sections of a normal (A-D) and regenerated mouse (E-H) TA post-femoral artery excision, immunostained for smooth muscle alpha-actin. A and E are registered using affine intensity based registration. B and F are registered using affine nucleus based registration. Within each column, the dashed lines indicate correspondence (according to color) between parts of the 2D sections and their locations on the 3D views. Also within each column, the lower case letter labels indicate correspondence between vessel cross sections on the 2D sections and their homologous locations within the 3D views. Blue arrows indicate incorrect vessel wall discontinuities arising from reconstruction error. The insets in the red boxes show 2D and 3D diameter measurements of the same vessel; note that the 2D measurement overestimates the 3D measurement by a factor of >6. Scale bars 100 μm.

Images of the 3D reconstructed volumes were rendered using 3D Slicer 4.1.1 (Harvard SPL, Boston, MA, USA), with a voxel size of 0.25 μm × 0.25 μm × 5 μm.[15] Regions of interest from whole-slide reconstructions of both normal and regenerated vasculature are shown in Fig 4, with selected corresponding 2D histological sections; both nucleus landmark-based and intensity-based reconstructions are shown. In Fig 4(C), a distinct vessel cross section is visible on one histology section, labeled as a. Fig 4(D) depicts another section taken 60 μm deeper into the tissue, showing two vessel cross sections, labeled as b and c. If only 2D histology images were available, one might conclude that these three vessel cross sections correspond to multiple distinct vessels. However, the 3D reconstruction shown in Fig 4(B) reveals that a, b, and c are in fact all connected within the same vessel. Fig 4(G) and (H) depict two sections taken 50μm apart, with seven vessel cross sections indicated with labels d through j. Based on inspection of only these 2D sections, one might conclude that there were four distinct vessels represented: one vessel d appearing on only one section, and three more vessels connecting e to h, f to i, and g to j, respectively. However, the 3D reconstruction of this tissue shown in Fig 4(F) reveals that in fact the connectivity is d to h, e to i, and an undulating vessel connecting f, j, and g. Thus, both the number of distinct vessels and their connectivity would be incorrectly estimated based on 2D sections alone. Additionally, the undulation creates the appearance of incorrectly large vessel lumina in 2D, which is demonstrated particularly for cross sections labeled f and j, arising from the fact that the plane of sectioning runs nearly parallel to the direction of the vessel in these areas. We compared spatially corresponding 2D and 3D manual diameter measurements of a total of 40 randomly chosen vessels from all 11 mouse samples. This yielded 40 paired differences (2D diameter— 3D diameter), the median ± interquartile range of which was 21.09 ± 27.95 μm. We also calculated the 2D diameter / 3D diameter ratio for each of the 40 vessels and found a median ± interquartile range of 1.6 ± 1.2. For each of the 40 vessels, the 2D diameter was larger than the 3D diameter.

#### 3.1.1 Registration technique comparisons

We observed qualitative differences between the different reconstruction approaches, with a smoother reconstruction in the nucleus landmark registration. Volumes depicting 3D reconstructions of normal and regenerated vasculature are shown in Fig 4. The intensity based reconstruction was generally observed to preserve vessel topology/connectedness for larger vessels but more geometric disruptions were qualitatively observed, compared to the nucleus-based reconstructions as seen in Fig 4(A) and 4(E) (intensity-based reconstruction), compared to Fig 4(B) and 4(F) (nucleus-based reconstruction), respectively.

### 3.2 Evaluation of registration accuracy

In all of our experiments, the TRE values were found to be non-normally distributed (*p* <. 01), therefore non-parametric testing (Wilcoxon sign rank test) of null hypotheses of equivalent medians was performed, and descriptive statistics were reported as the median ± interquartile range (IQR). For reference in terms of the best possible performance using non-rigid affine registration, the measured pairwise FRE and the TRE of the reference reconstruction are shown in Table 1. Table 2 shows both the pairwise and accumulated TRE values for normal and regenerated samples, with 95% confidence intervals on the medians. Intensity-based registration at low resolution was also reported, which was used to initialize the subsequent high-resolution intensity- and nucleus landmark-based registrations. Table 2 also shows the pairwise and accumulated TRE values for these subsequent rigid and non-rigid affine registrations for both high resolution intensity based registration and nucleus landmark registration. The intensity based registrations were performed on grayscale images; no significant differences were found between these reported error values and those measured when the registration was performed on the red, green or blue channels separately. Since the TRE values were found to be non-normally distributed, these confidence intervals were computed non-parametrically as *[L*, *U]*, with lower bound *L* and upper bound *U* defined as the TRE values having ranks of $n/2-1.96\;\sqrt{n}/2$ and $n/2+1.96\;\sqrt{n}/2$, respectively, in the sorted list of TREs. [16] There was a statistically significant difference in the median pairwise TRE values between the intensity-based and the nucleus-based registrations for normal and regenerated mouse tissues (*p* <. 001). The medians of the accumulated TRE were also statistically significantly different between the intensity-based and the nucleus-based registrations for both normal and regenerated tissues (*p* <. 001). For both normal and regenerated tissues, we also calculated the maximum post-registration distance between any pair of homologous nucleus landmarks used for the TRE calculation. Table 3 reports the mean and standard deviation of these maximum differences.

**Table 1:** Pairwise affine registration errors (μm) of the reference nucleus landmarks.

Normal	Median ± IQR	CI 95%
**FRE**	3.42 ± 4.44	[3.32,3.52]
**TRE**	3.54 ± 4.63	[3.42,3.63]
**Regenerated**		
**FRE**	2.49 ± 4.44	[2.43,2.57]
**TRE**	2.57 ± 4.63	[2.50,2.65]

*FRE: fiducial registration error, TRE: target registration error

**Table 2:** Pairwise and accumulated target registration error (TRE) values (μm) of the rigid and affine intensity-based and nucleus-based landmark registration (best in boldface).

		Rigid Registration		Affine Registration	
		Normal	Regenerated	Normal	Regenerated
**Pairwise TRE (μm)**					
Intensity-based (low-res)	Median ± IQR	12.90 ± 13.85	10.31 ± 13.03		
	CI	[12.6,13.3]	[10.1,10.6]		
Intensity-based (high-res)	Median ± IQR	9.07 ± 14.96	6.70 ± 7.39	7.70 ± 12.41	4.73 ± 6.33
	CI	[8.81,9.34]	[6.53,6.89]	[7.43,7.99]	[4.61,4.87]
Nucleus based	Median ± IQR	**6.54** ± 8.02	**5.93** ± 7.68	**4.97** ± 5.75	**4.54** ±6.67
	CI	**[6.36,6.76]**	**[5.74,6.07]**	**[4.87,5.12]**	**[4.41,4.68]**
**Accumulated TRE (μm)**					
Intensity-based (low-res)	Median ± IQR	34.43 ± 42.49	31.58 ± 32.70		
	CI	[33.4,35.6]	[30.8,32.4]		
Intensity-based (high-res)	Median ± IQR	33.70 ± 40.48	17.95 ± 23.71	22.49 ± 38.59	13.62 ± 20.19
	CI	[32.8,34.7]	[17.3,18.5]	[21.7,23.2]	[13.1,14.1]
Nucleus based	Median ± IQR	**12.67** ± 15.84	**12.42** ± 12.91	**9.78** ± 17.09	**9.81** ± 15.71
	CI	**[12.3,13.1]**	**[12.0,12.7]**	**[9.4,10.2]**	**[9.4,10.1]**

**Table 3:** Mean and SD of maximum pairwise and accumulated target registration error (TRE,) observed on each section for the rigid and affine intensity-based and nucleus-based landmark registration (best results in boldface).

	Pairwise (Mean ± SD)		Accumulated (Mean ± SD)	
	Normal	Regenerated	Normal	Regenerated
**Rigid Registration TRE (μm)**				
Intensity-based (low-res)	49.02 ± 32.19	31.73 ± 17.65		
Intensity-based (high-res)	47.47 ± 38.51	26.96 ± 19.26	46.47 ± 35.40	**24.06** ± 15.34
Nucleus based	**38.89** ± 30.68	**24.32** ± 15.43	**35.87** ± 32.06	24.49 ± 21.67
**Affine Registration TRE (μm)**				
Intensity-based (low-res)	90.22 ± 52.86	66.22 ± 38.74		
Intensity-based (high-res)	90.87 ± 79.05	37.14 ± 22.20	78.63 ± 65.44	41.78 ± 25.31
Nucleus based	**43.06** ± 34.71	**28.47** ± 12.80	**30.05** ± 23.52	**39.41** ± 48.03

**Fig 5:**
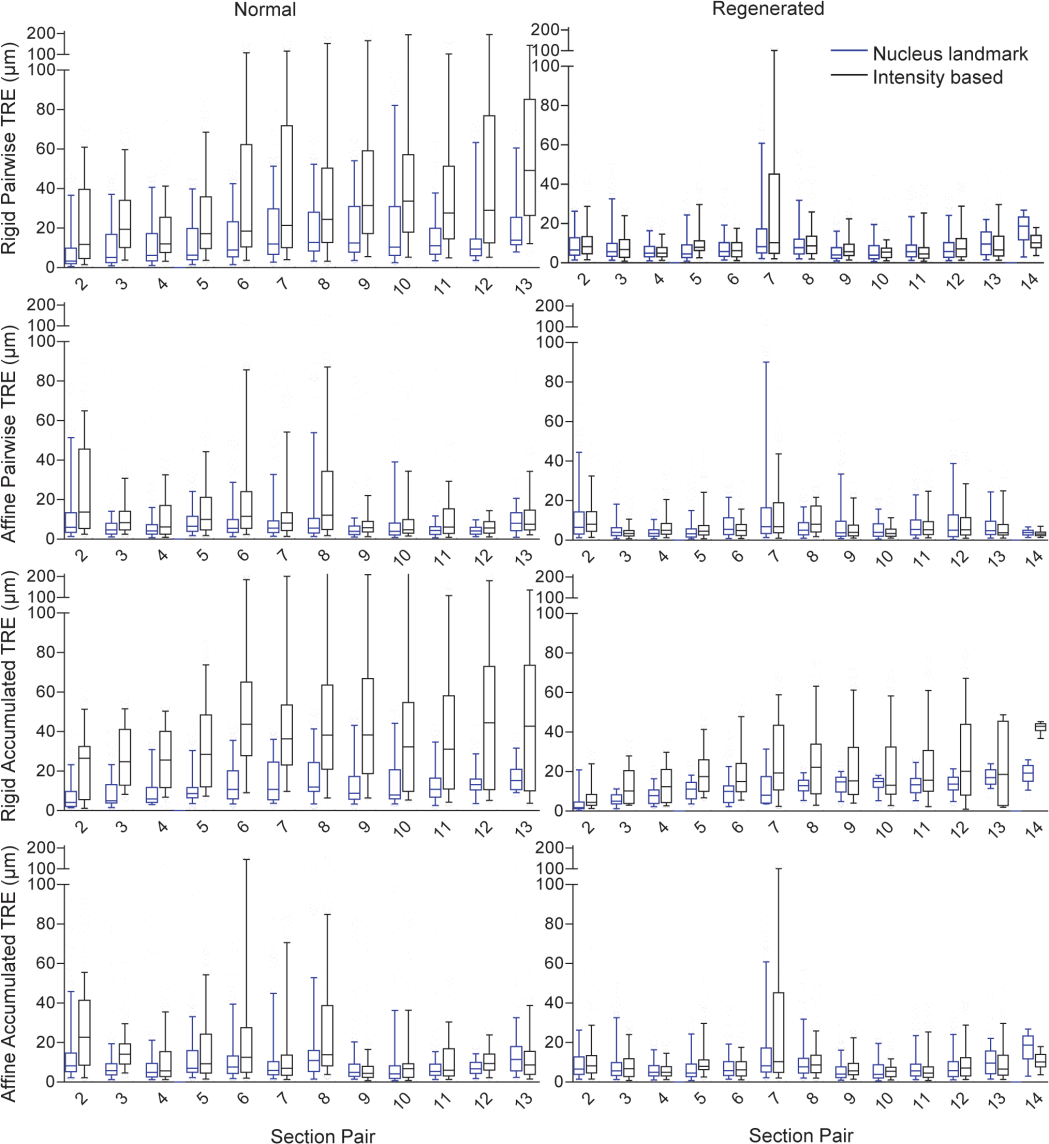
Registration accuracy measurement values. Box plots of the rigid and affine target registration error (TRE) computed for each adjacent pair of sections (pairwise) and propagated throughout the 3D reconstruction (accumulated).

Box plots of the distributions of the TRE values are shown in Fig 5 for the high resolution affine intensity based registration and the affine nucleus landmark based registration. These plots provide a more detailed view of the error measurements, and showing the distributions of pairwise and accumulated TRE values at every section in the reconstruction. For the pairwise TRE, Fig 5 shows the TRE distributions for each section pair, where the number on the horizontal axis indicates the larger of the two section numbers in the pair (e.g. at horizontal axis point 2, the pairwise TRE distribution for sections 1 and 2 is shown). For the accumulated TRE, Fig 5 shows the TRE distributions for each section number as indicated on the horizontal axis. For the pairwise TRE, the horizontal axis value of one clearly has no defined TRE distribution, and for the accumulated TRE at this point on the horizontal axis all TREs are zero (since section 1 is an untransformed reference section forming the basis of the reconstruction). It is for this reason that the horizontal axes begin at section 2 in Fig 5. For the rigid transformation model, we observed a more pronounced trend toward increasing accumulated TRE values with increasing section number for the intensity-based reconstruction, as compared with the nucleus-based reconstruction. For most sections, concordant with the observations in Tables 2 and 3, we observed larger error magnitude and variability using intensity-based registration. Overall, the affine transformation model outperformed the rigid transformational model and suffered fewer effects of accumulation of error.

### 3.3 Evaluation of image similarity metrics

Table 4 shows the medians and interquartile ranges of the displacements described in the experimental methods along the x and y directions for normal and regenerated samples, and for all samples in aggregate. The upper bounds of the associated prediction intervals are also shown. As these samples were found to be non-normally distributed, a non-parametric approach to calculating the prediction interval was used, where the maximum TRE value observed in the sample defined the upper bound of the *P* % prediction interval, where *P* = (*N* − 1) /(*N* + 1). [17] For all the samples in aggregate, an upper bound of not more than 10 μm was observed in the 98% prediction interval, suggesting that with robust optimization of the MSE image similarity metric on full resolution images, the resulting pairwise registrations are unlikely to be displaced more than 10 μm from those given by the nucleus-based registration.

**Table 4:** The displacement of the optimal mean squared error transformation from the affine nucleus-based registration.

Sample	Displacement direction (μm)	Median ± IQR	Upper bound of non-parametric P% prediction interval (μm)	P (%)
**Normal**	X	1.50 ± 2.25	9.58	97
	Y	1.00 ± 1.50	5.37	97
**Regenerated**	X	1.50 ± 2.00	6.50	97
	Y	1.50 ± 2.00	8.87	97
**All Values**	X	1.50 ± 2.38	10.00	98
	Y	1.50 ± 1.50	8.52	98

## Discussion

Vascular abnormalities perturb organ perfusion and could lead to damage and tissue dysfunction. The microvasculature which underlies tissue perfusion is inherently 3D and aspects of the vascular pathology may be unaccounted for when assessing the vessels in conventional 2D histology. Visualizations in 3D could remove potential ambiguities in the interpretation of the histological samples. This is demonstrated by our results in Fig 4, where several different types of misinterpretations may potentially occur. The density of vessels in one region could be misinterpreted and the branching or structural detail at the bifurcation point is lost. The reconstructed normal tissue in (B) demonstrates the connectivity of the vessels shown in the cross sections (C) and (D). Without the 3D image, the interpretation of the connectivity of microvasculature can be challenging. Cross sections 50 μm apart in Fig 4(G) and 4(H) demonstrate regenerated vessels, portions of which (indicated by f and i) run nearly parallel to the section plane. In this example, the vessels could be misinterpreted to have larger lumina and have a higher vessel count in plane due to multiple views in cross section of one vessel. For example, the vessel labeled f in Fig 4(G) is connected to the vessel labeled g on the same figure due to the tortuosity of the vessel. When quantifying vessel features within 2D sections, this tortuosity could lead to an erroneously high vessel density count. In the corresponding reconstruction (F), connectivity, tortuosity and thickness of the vessel are visualized. Errors in 2D measurement of vessel diameter arising from non-orthogonality of vessels to sections can be resolved in a 3D reconstruction. This is demonstrated with an example in Fig 4, where the measured lumen diameter in 2D is 93 μm due to the vessel running nearly parallel to the section plane. In the 3D reconstruction, we are able to measure the diameter of the vessel in a direction perpendicular to the vessel’s 3D centerline, yielding a lumen diameter of 15 μm. Our experiment comparing 2D and 3D measurements on 40 different vessels revealed that 2D measurements are biased to overestimate vessel diameter, with overestimations of 50% or more occurring frequently. These observations are clear in the 3D reconstruction but nearly impossible to make with only the ambiguous 2D histology.

Previous work on 3D imaging of vessel wall components has been performed using phase-contrast computed tomography (CT) imaging in order to differentiate the soft tissue layers of the human carotid artery and potentially characterize arterial plaque. [18] The authors showed that several layers of formalin-fixed carotid artery could be differentiated using a conventional X-ray tube with 100 μm resolution, using radiation doses much higher than those used for clinical CT imaging. Although 100 μm resolution is insufficient for microvasculature visualization (where vessel sizes are approximately 10 μm and vessel wall components are even smaller), our 3D histology reconstruction approach could be used to address the authors’ stated difficulty in obtaining a precise co-registration between their 2D histologic sections (used for CT imaging validation) and the 3D CT images. Our approach is also complementary in its ability to provide 3D reconstructions of specific structures and proteins revealed by histology stains, complementing the vessel wall layer information provided by phase-contrast CT.

These recent investigations make clear the need for highly accurate, automated, repeatable 3D reconstructions of thin histology sections both as a complement to confocal microscopy and as an independent means of answering important research questions involving microvasculature analysis and quantification. The automatic registration of the serial sections would speed up the process of manual alignment for volume reconstruction by increasing the sample size with a minimal corresponding increase in manual labour and time. The smooth muscle surrounding the vasculature was stained using the smooth muscle α-actin primary antibody in the samples of this study, but other tissue components could also be stained and incorporated into the 3D reconstruction. Multiple stains could be applied to the tissue to visualize different components; e.g., vascular endothelial cells and smooth muscle could be stained to visualise both components. [19] The reconstruction of histology into the 3D context could also facilitate co-registration with 3D in vivo imaging (as has been done at the millimeter scale in human imaging [20–22]) such as micro-CT, where co-registered 3D histology could provide complementary information to the vessel lumina visualized using the iodinated CT contrast agent.

In this study, we evaluated intensity-based and nucleus landmark-based 3D reconstruction methods on TA muscle samples of wild type mice under both normal and hind limb ischemic conditions. Our hybrid approach uses a coarse initialization from on a low-resolution intensity-based registration, followed by a refinement using a landmark-based registration defined using bisected cell nuclei appearing on adjacent tissue sections. Our approach is inspired by previously published methods that applied intensity-based coarse registration followed by landmark-based registration refinement, [4] but differs from previous work in two primary respects. First, in contrast to methods relying on the introduction of extrinsic landmarks (e.g. needles) into the tissue, [23] our approach automatically extracts and corresponds intrinsic cell nuclei, resulting in a more streamlined workflow and reduced tissue disruption. Second, our specific choice of landmark type (cell nuclei) differs from landmark types used in previous work and has demonstrated high accuracy for 3D reconstruction both in terms of pairwise and accumulated TRE. This is based on the observation that the accuracy of a 3D reconstruction using landmark-based registration depends on the characteristics of the landmarks used to define the registration transformation. This issue is especially important in the context of a 3D reconstruction that is defined as a composition of multiple pairwise registrations; any spatial bias in pairwise landmark correspondence error can propagate through the pairwise registrations and result in a large accumulated error in the reconstruction. Our choice of small cell nuclei as landmarks mitigates this issue as nucleus orientation in our specimens would not be expected to have a spatial bias that would contribute to such error propagation. It is for this reason that our method can overcome the issue of error propagation and the banana-into-cylinder effect, as evidenced by our reported accumulated TRE values. Due to the abundance of nuclei in the tissue, the registration is robust to the accuracy of segmentation of the cell nuclei when the majority of nuclei present are segmented (as is the case using our algorithm). The number of nucleus landmark correspondences obtained by our algorithm far exceeds the number of pairs necessary for defining an affine or rigid registration.

Our interest in ischemia and reperfusion is due to the critical need to understand the regeneration process of the vasculature, as this has important therapeutic potential for patients with vascular disease. However, this potential has yet to be fully realized and understanding the 3D relationships amongst the neovasculature and the surrounding ischemic and/or regenerating tissue will be important in advancing this field. [24] We anticipate that in a larger study of these mice, renderings of 3D reconstructed histology will clearly show the microvasculature connectivity at the level of the arterioles and venules, which would not be apparent in conventional 2D histology analysis (Fig 4). We also anticipate that 3D histology reconstructions will permit more reliable quantifications of important surrounding tissue components that can affect tissue perfusion, such as the volume and surface area of the nearby skeletal muscle fibers.[25] In addition, vessel surface area cannot be measured in 2D histology, whereas it is straightforward to measure in 3D on a segmented vessel network. As another illustration, due to variability in the angle of sectioning, the medial wall thickness and vessel diameter can be difficult to interpret. For example, if the tissue was sectioned off-orthogonal to the vessel, the lumen would likely be measured to have a larger area than the correct area that would be measured if the tissue were sectioned orthogonal to the vessel direction (Fig 4G). We speculate that vascular measurements, such as the vessel wall thickness, may be more consistent in 3D, especially when malformations cause structural changes.

### 4.1 Evaluation of registration accuracy

It is helpful to the interpretation of registration errors in comparison to the reference reconstruction to note that the lower bound of an arteriole microvessel diameter in the mouse is approximately 10 μm. [26] Imaging the tissue prior to sectioning may generate a volume which does not have resolution high enough to resolve microvascular structures, thus a surrogate reference was used. The reference reconstruction generated through manually validated, intrinsic nucleus landmarks provides a surrogate for an ideal 3D reference volume, where the intrinsic landmarks are not subject to orientation bias. The pairwise and accumulated TREs indicate that in the normal and regenerated tissue, the 95% confidence intervals on the median TRE were lower than 5.2 μm for the affine nucleus landmark registration (Table 2), which suggests accurate reconstruction of both microvessel topology and geometry in these samples. The reference FRE shown in Table 1 provides a sense of the best-case registration that can be achieved using an affine transformation of the manually validated reference landmarks; the FRE thus provides insight into the amount of residual deformation in the tissue.

The intensity-based reconstruction did not provide 95% confidence intervals of less than 10 μm for the accumulated TRE values. This, combined with the observation that the nucleus-based reconstruction provided lower accumulated TRE for both the normal and the regenerated tissues, suggests that this nucleus landmark-based registration is valuable for 3D histology reconstruction of microvasculature. To place our results within the context of a comparable, recently published method investigating the accuracy of 3D histology reconstructions based on semi-automatic non-rigid B-spline registration [10], we calculated and compared results analogous to those presented in [10] (Table 3). The authors of [10] demonstrated reconstructions of metastatic colorectal carcinoma in human liver tissue, cirrhotic human liver tissue infected with hepatitis C, and a rat glomerulus. Reconstruction error was quantified in terms of Hausdorff distance, and reported mean ± SD pairwise Hausdorff distance errors of 49 ± 31 μm and 54 ± 37 μm respectively for two specimens. Corresponding reported mean ± SD accumulated Hausdorff distance errors (over 10 sections) were 112 ± 71 μm and 120 ± 88 μm, respectively. By comparison, for our fully-automatic *affine nucleus-based landmark* registration, we observed mean ± SD accumulated maximum per-section TREs of 30 ± 24 μm and 39 ± 48 μm for normal and regenerated tissues respectively (Table 3).

The authors of [6, 27] proposed an intensity-based registration approach for 3D histology reconstruction of tumor microvasculature using iterative optimization of an image similarity metric to align a series of adjacent section pairs; our tested intensity-based method is similar in these respects. The accuracy of the registrations in [6, 27] was evaluated by the authors using qualitative inspection of the reconstructed volumes; our work extends this work by quantitatively measuring the registration error using intensity-based registration. The method described in [28] intended for alignment of individual cells used a multi-resolution intensity-based registration. On their data sets consisting of serial sections, the majority of registration errors were between 10 and 30 μm; this is concordant with our mean intensity-based registration errors (Table 2). The method described in [29] is intended for human liver tissue reconstruction used a block-matching intensity-based registration approach to align sections with the same type of stain. Their results presented Hausdorff distance errors most frequently lying between 50 and 150 μm on a single specimen, which is concordant with our maximum observed intensity-based registration errors (Table 3). Although the work presented in [30] was not intended for microvasculature reconstruction, it has similarities to our work in that structures of interest are segmented and used to refine an initial intensity-based registration for reconstruction of mammary gland tissue. Reconstruction accuracy on real image data in [30] was assessed by qualitative inspection of the 3D volumes. Similarly, the accuracies of the feature-based reconstruction methods proposed in [4, 7, 31] were not measured quantitatively on real image data, challenging the assessment of their utility for microvasculature reconstruction and analysis where the requirements for accuracy are stringent. A block matching method proposed in [14] reported a pairwise root mean square error (RMS) of 23.25 μm, which is larger than our pairwise nucleus landmark-based registration RMS TRE values of 14.25 μm. Our work extends these investigations by providing a rigorous quantitative assessment of reconstruction error for a feature-based registration approach in the context of microvasculature assessment in the mouse.

As intensity-based registration is driven by optimization of an image-similarity metric computed over the entire image domain, misalignment of large, salient structures produces a larger penalty to the optimizer and thus the tendency is to provide a registration aligning such structures from one section to the next. Registration of salient structures may propagate pairwise error through the reconstructed volume, and this pairwise error may appear negligible unless the salient structures have spatial bias. One example could be due to a large blood vessel oriented non-orthogonally to the section. The result can be a non-negligible accumulation of error throughout the reconstructed volume. This accumulation of registration error across multiple sections has been previously observed in other intensity based pairwise registration methods. [4, 10, 29] Knowledge of accurate structural orientation can be vital to differentiation between pathological models. These geometric measurements could be perturbed by pairwise registration of salient features, which may force non-orthogonal features to be straight and section-orthogonal. In the hind limb skeletal muscle tissues, the vasculature flows parallel to muscle fibers,[32] and the tissue sections were taken in the transverse plane. In this case, forcing the salient muscle fibres to align pairwise and run orthogonal to the plane of sectioning may have been expected to result accurate 3D reconstructions. However, the accumulated TRE values (Table 2) and the reconstructed volumes (Fig 4) for the intensity-based registration indicate otherwise. This accumulation of error may pose an even greater problem in tissues with salient features which are not orthogonal to the plane of sectioning, such as muscle fibers in the heart which have a helical configuration. Our results suggest that landmark-based registration of small features such as cell nuclei may aid in the alignment of tissues where geometry preservation is a priority.

The non-negligible accumulation of error in the reconstructed volumes could lead to vessel structure misinterpretations. As seen in Fig 4, although the differences in intensity-based and landmark-based registration of the adjacent sections are not visually salient, the impact of these differences is clear in the 3D reconstructions of the normal and regenerated mice. The bias in the intensity based registration to align large, salient features on the adjacent sections is disruptive to the preservation of microvasculature continuity.

### 4.2 Evaluation of image similarity metrics

Based on the upper bound of the 95% prediction interval across all three samples, global MSE optimization would transform the moving image to be not more than 10 μm—100% of the typical diameter of a microvessel—from the transformed moving image given by nucleus-based registration. It could therefore be expected that such differences could be disruptive to useful 3D histology reconstructions for microvasculature analysis. This suggests that this image similarity metric may not be suitable for this problem. In our informal initial experiments, we did not observe reduced error using the normalized cross correlation or mutual information image similarity metrics, so we opted to test the MSE metric in the interests of computational efficiency for this mono-modality registration problem. The use of MSE seems particularly appropriate given that each of our samples was processed in one preparation to mitigate any potential effects of staining variability, with variables such as the concentration of the solutions and the incubation time controlled.

### 4.3 Limitations

The scope of this study was to perform a technical evaluation of intensity-based and nucleus landmark-based registration algorithms on the two types of mice: those with normal vasculature, and those with post-ischemic regenerated vasculature. The differences observed between the nucleus landmark and intensity based registrations were consistent with both the normal and regenerated vasculature, with consistently higher accuracy using the nucleus landmark registration technique. Comparison of vessel structure in the two types of mice is of interest and the subject of future work; the work presented in this paper is a critical first step toward this future aim.

The results of this study need to be considered in the context of its strengths and limitations. Although two distinct tissue types were studied, the sample size is small and thus new insights may be expected to arise with additional samples. Moreover, the approach can be adapted for variation in anatomy, pathology or stain. The key observation was the use of small structures not spanning more than two adjacent sections could reduce the banana-into-cylinder problem without the use of costly equipment. Serial sections cut by the microtome can be technically challenging, but in the case of missed sections, a fine scale alignment of the tissue conserving structural context can be used for exploration of the overall tissue. The local neighbourhood size *T* = 100 μm was chosen (based on experimentation with a sample not included in this study) to encompass the observed pairwise TREs from the intensity-based registration, in an effort to ensure that corresponding nuclei lay within this neighbourhood. However, this parameter represents a brittle aspect of our algorithm; if a correctly corresponding nucleus is outside of this range, correct correspondence cannot occur. Our informal experimentation suggests that the algorithm is not particularly sensitive to this neighbourhood size, but nevertheless this is an important parameter that may need to be modified when adapting this algorithm to other contexts, according to the observed error in the intensity-based registration. Finally, our conclusions regarding the performance of intensity-based registration for this task must take into account the limitations of our experimental design; we tested only the MSE image similarity metric and used gradient descent optimization. Although our results demonstrated that ideal optimization of the MSE metric would yield reconstructions different from those given by our nucleus landmark-based approach, we cannot conclude from this that a more suitable image similarity metric (e.g. an approach based on M-estimators [14]) could not be devised that would be suitable for this problem, and this would be a useful avenue of future work in this area.

## Conclusions

We have demonstrated that accurate 3D reconstructions of serial histology sections of mouse hindlimb tissue can avoid potential misinterpretations of the vasculature arising from assessment of 2D histology sections without 3D context. Such misinterpretations may relate to vessel size, tortuosity, connectivity, and bifurcation, all of which are important to understand pathologies of the vascular network at the arteriolar and venular levels. Our results demonstrated that a 3D reconstruction algorithm based on section pairwise registrations using small, homologous landmarks spanning not more than two tissue sections may support 3D reconstruction of digital histology images with sufficient accuracy to provide acceptable registration for the arterioles and venules. This technique avoids the otherwise problematic “banana-into-cylinder” effect where conventional intensity-based registration methods optimize the pairwise alignment of large, salient structures, forcing them to be section-orthogonal. Our results demonstrated that 3D digital histology reconstruction of mouse hind-limb tissue stained for vascular smooth muscle cells and hematoxylin could be performed accurately and fully automatically via a cascaded approach beginning with pairwise low-resolution intensity based registration of adjacent tissue sections, and refined by a landmark-based registration using corresponding nuclei that were bisected by the microtome blade during sectioning. Intensity-based reconstructions driven by larger, more salient features appear to preserve vascular topology but not geometry; the use of nuclei for refinement of the reconstruction achieves both ends. With our ongoing validation and refinement of this system on a larger data set, we aim to provide a valuable tool for scientists conducting studies requiring high-throughput, high-accuracy (<10 μm error) 3D histology reconstructions for analysis of 3D microvasculature and surrounding tissue components in small animal models.

## Supporting Information

S1 Dataset Affine Raw Values. Raw target registration error values in microns for each sample and each section for accumulated nucleus based registration.
(XLSX)

S2 Dataset Fiducial Raw Values. Raw fiducial registration error values in microns for each sample and each section of the pairwise fiducials.
(XLSX)

S3 Dataset Rigid Raw Values. Raw target registration error values in microns for each sample and each section for pairwise intensity based low resolution registration.
(XLSX)

S4 Dataset Translate Raw Values. Raw mean squared error values of translation in the X direction for each sample between each adjacent section pair.
(XLSX)

S1 Fig Regenerated hind limb tissue following ischemic damage. The tissue was immunostained with smooth muscle α-actin and DAB chromogen and counter-stained with hematoxylin. Blue arrows indicate nuclei and black arrows indicate the stained arteriole. Scale bar (A) 500μm, (B) 100μm.
(EPS)

S2 Fig Separate set of mouse hind limb tissues that were not used for the experiments reported in this paper. These tissues were used to determine nucleus size and color for nucleus extraction. Scale bar 100 μm.
(EPS)

S3 Fig Nucleus segmentation method. (A) The segmented nucleus labeled blue on the histology section stained with DAB and hematoxylin counter stain. (B) The surrounding region (white) of each nucleus was defined by morphological dilation and used to evaluate whether the extracted nucleus was within the tissue, or was instead a false positive corresponding to debris on a white background outside of the tissue, indicated with the white arrows. Scale bar 50 μm.
(EPS)
